# 

**Published:** 1888-01

**Authors:** 


### BOOKS RECEIVED.

Hydrophobia—An Account of Pasteur's System. By Reynaud Suzor, M. B., C. M. Edin., M. D. Paris.

The Urine. Memoranda Chemical and Microscopical for Laboratory Use. By J. W. Holland, M. D.

Manual of Clinical Diagnosis. By Dr. Otto Seiffert and Dr. Freidreich Müller. Third Edition. Translated by William B. Canfield, A. M., M. D. Berlin.

On the Animal Alkaloids—The Ptomäines, Leucomäines and Extractives, in their Pathological Relations. By Sir William Aitken, Knt., M. D., F. R. S.

Lectures on Diseases of the Brain. By William R. Gowers, M. D., F. R. S.

The Principles and Practice of Medicine. Volume II. By Charles Hilton Fogge, M. D., F. R. C. P.

Transactions of the American Surgical Association. Volume V. Edited by J. Ewing Mears, M. D.

A Practical Treatise on Materia Medica and Therapeutics. By Roberts Bartholow, A. M., M. D., LL. D.

Six Hundred Medical Don'ts. By Ferd. C. Valentine, M. D.

The Throat and its Diseases. By Lenox Browne, F. R. C. S., E.

A Manual of Organic Materia Medica. By John M. Maisch, Phar. D.

Text-Book of Therapeutics and Materia Medica. By Robert T. Edes. A. B., M. D.

The Study of History in American Colleges and Universities. By Herbert B. Adams, Ph. D.

Health Lessons—A Primary Book. By Jerome Walker, M. D.

Anatomy, Descriptive and Surgical. By Henry Gray, F. R. S., etc.

Volume LVI.] JANUARY, 1888. [Number 1.

### The CHICAGO MEDICAL JOURNAL AND EXAMINER

EDITOR:
S. J. JONES, M.D., LL.D

COLLABORATORS:

CHICAGO:

PROFESSOR E. ANDREWS, M.D., LL.D
PROFESSOR J. BARTLETT, M.D.
PROFESSOR W. T. BELFIELD, M.D.
PROFESSOR F. BILLINGS, M.D.
PROFESSOR W. H. BYFORD, A.M., M.D.
PROFESSOR L. CURTIS, A.M., M.D.
PROFESSOR N. S. DAVIS, JR., A.M., M.D.
PROFESSOR O. C. DEWOLF, A.M., M.D
PROFESSOR E. C. DUDLEY, A.B., M.D.
PROFESSOR C. FENGER, M.D.
PROFESSOR J. N. HYDE, A.M., M.D.
PROFESSOR E. F. INGALS, A.M., M.D.
PROFESSOR F. S. JOHNSON, A.M., M.D.
PROFESSOR H. A. JOHNSON, M.D., LL.D.
PROFESSOR C. W. PURDY, M.D.
PROFESSOR C. G. SMITH, A.M., M.D
PROFESSOR E. WING, A.M., M.D

MILWAUKEE, WIS.
PROFESSOR N. SENN, M.D

WASHINGTON, D. C.:

S. O. RICHEY, M.D.

UNITED STATES ARMY:
SURGEON D. L. HUNTINGTON, M.D.

UNITED STATES NAVY:
MEDICAL DIRECTOR J. M. BROWNE, M.D.
MEDICAL DIRECTOR A. L. GIHON, A.M., M.D

MARINE HOSPITAL SERVICE:
SURGEON S. T. ARMSTRONG, M.D.

